# Custom-Made Dual-Functional Oral Appliance for Management of Obstructive Sleep Apneic Completely Edentulous Patient

**DOI:** 10.7759/cureus.16656

**Published:** 2021-07-27

**Authors:** Fathima Banu, Karthigeyan Jeyapalan, Anand Kumar V

**Affiliations:** 1 Prosthodontics, Faculty of Dental Sciences, Sri Ramachandra Institute of Higher Education and Research, Chennai, IND

**Keywords:** sleep apnoea, mandibular advancement device, osa, sleep disorder, tongue protrusion device

## Abstract

Continuous positive airway pressure (CPAP) being a gold standard treatment to open the upper airway by application of controlled compressed air is still not a widely accepted mode of treatment among obstructive sleep apnea (OSA) individuals. To improve patency of upper airway space and reduce the risk of sleep apnoea, it is essential to provide mandibular advancement devices (MADs) that could provide non-continuous positive airway pressure (non-CPAP) for patients with OSA. Availability of prefabricated oral appliances (OAs) like MADs, tongue holding devices reduced the chair-side fabrication time but has poor adaptation, excessive salivation, and deprivation of sleep. Customized OAs can overcome these challenges, but their fabrication for an edentulous individual is challenging due to the absence of teeth and the encroachment of tongue space by the device. This clinical report gives an insight into the clinical and technical aspect of fabrication of MAD with tongue retaining space for an edentulous individual with OSA.

## Introduction

Obstructive sleep apnea (OSA) is the most frequent causative factor for the sleep disturbances in an adult population due to the alteration in the size and tone of the pharyngeal musculature resulting in the complete or partial collapse of the pharyngeal walls [1]. The condition is accentuated by the loss of vertical dimension in completely edentulous patients which reduces the upper airway space [2]. Majority of population affected with OSA have repetitive and intermittent cessation of breathing that is often reflected as snoring [3]. Individuals with OSA also have an inadequate oxygen supply due to apnea which increases the blood carbon dioxide pressure and eventually awakens the patient momentarily, to breathe in, and return to sleep without consciously remembering the episode [4]. Though the affected individuals do not commonly report their difficulties in breathing, they are most commonly identified from the complication associated with untreated OSA such as cerebrovascular disease, diabetes mellitus, impaired cognitive functions, and depression [1]. An early identification and intervention of OSA can uplift their well-being and provide them a better quality of life. To reduce the risk of sleep apnea and increase the upper airway size, it is essential to provide mandibular advancement devices (MADs) that could provide non-continuous positive airway pressure (non-CPAP) [5]. However, fabrication of MAD for an edentulous individual is a challenging task due to the compromise in the retention and encroachment of tongue space by the prosthesis narrowing the posterior airway space [2]. This technical report gives an insight into the clinical and technical aspect of fabrication of a modified MAD for an edentulous individual with OSA.

## Technical report

Case description

A 45-year-old male patient suffering from OSA and non-compliance with CPAP therapy with disturbed night sleep and day-time drowsiness was referred from the Department of Otolaryngology to the Department of Prosthodontics for treatment. Intra-oral examination of the patient presented a completely edentulous maxillary and mandibular arches rehabilitated with removable dental prosthesis. To fabricate a MAD, master impressions of the denture bearing areas were made and occlusal rims were fabricated. Maxillary and Mandibular jaw relationship was recorded with occlusal rims on the trial denture base. Niswonger’s method was used to determine the vertical dimension at rest and a lateral cephalogram was taken to evaluate the posterior airway space. Based on the clinical and radiographic data, a custom-designed MAD with tongue retentive aid was planned over the edentulous arch.

Technical report

**Figure 1:**
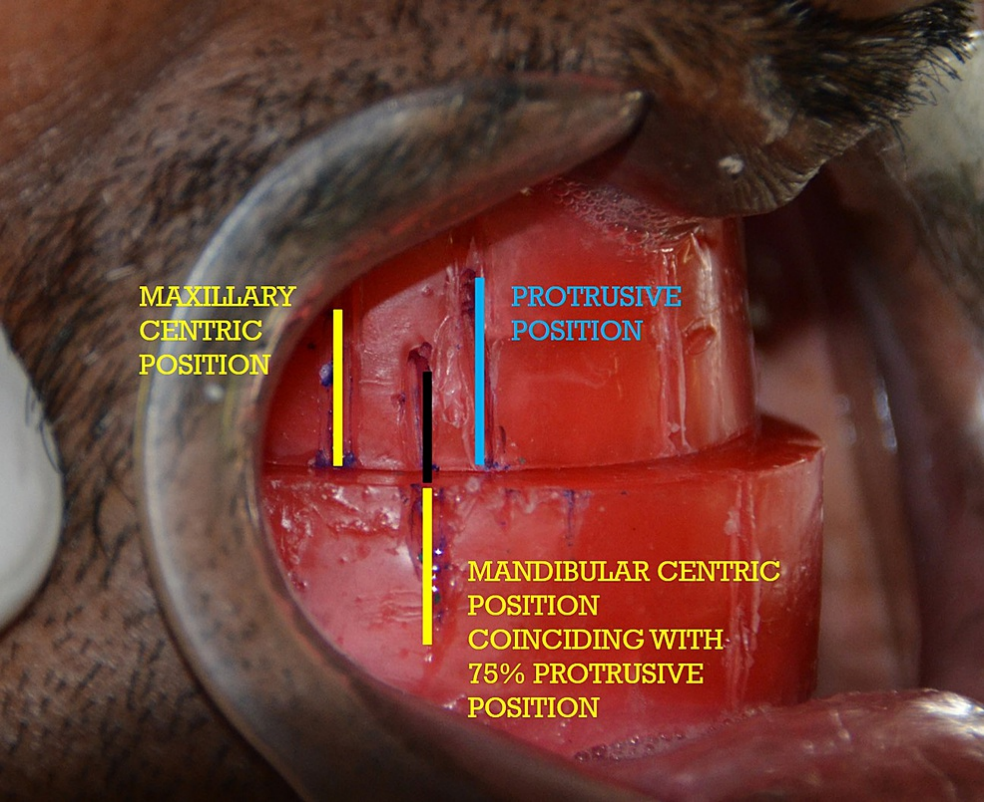
Maxillomandibular occlusal rims fused at 75% protrusive position

The centric relation and maximum protrusion positions of the patient were identified with the maxillary and mandibular occlusal rims using a popsicle stick. The patient was instructed to protrude the mandible at 75% of maximum protrusion and the occlusal rims were fused together as shown in Figure 1.

**Figure 2:**
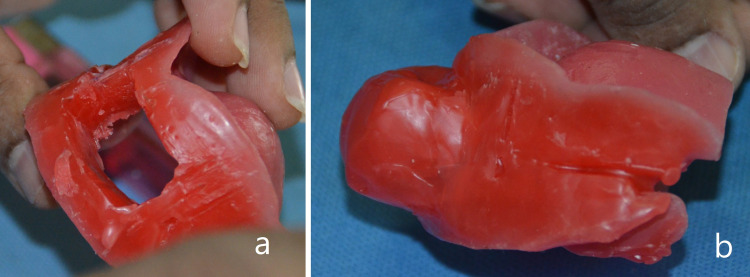
Wax removed from the anterior region (a) and bulb created (b) to provide space for protrusion of tongue

The portion of wax in the anterior region of the fused occlusal rims was removed (Figure 2a), and a wax bulb was fabricated to record the tongue in an anterior position (Figure 2b).

**Figure 3:**
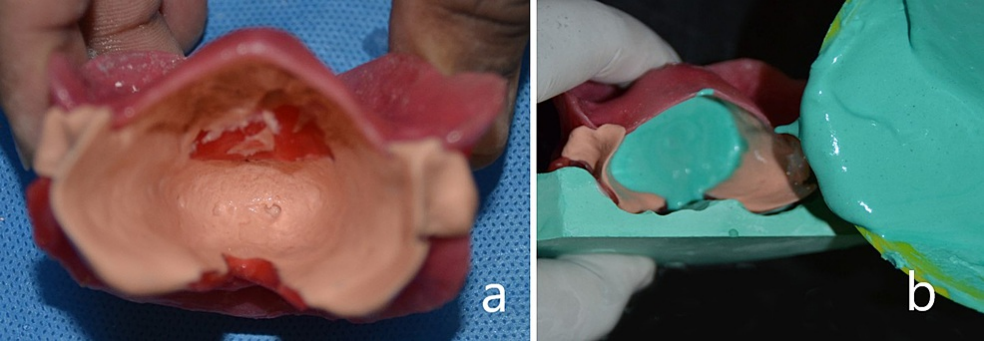
Impression of tongue in protrusive position (a) and stone mold poured in tongue space (b)

**Figure 4:**
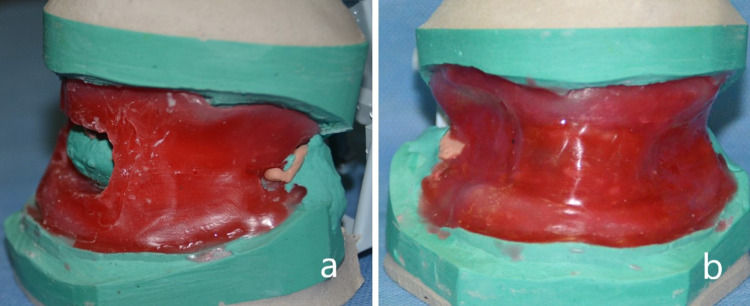
Tongue replica incorporated in the model (a) and completed wax-up of prosthesis (b)

Irreversible hydrocolloid with thin consistency was placed into the tongue space region and the patient was instructed to protrude the tongue (Figure 3a). After recording the impression of the tongue, the bite rims were retrieved as one unit and positioned over the maxillary and mandibular casts. The tongue space mould was poured using dental stone (Figure 3b). The tongue replica was integrated with the mandibular cast and was articulated using a hinge articulator with a posterior vertical stop (Figure 4a). The wax-up for the MAD was completed and maxillary-mandibular casts were de-articulated as a single unit (Figure 4b).

**Figure 5:**
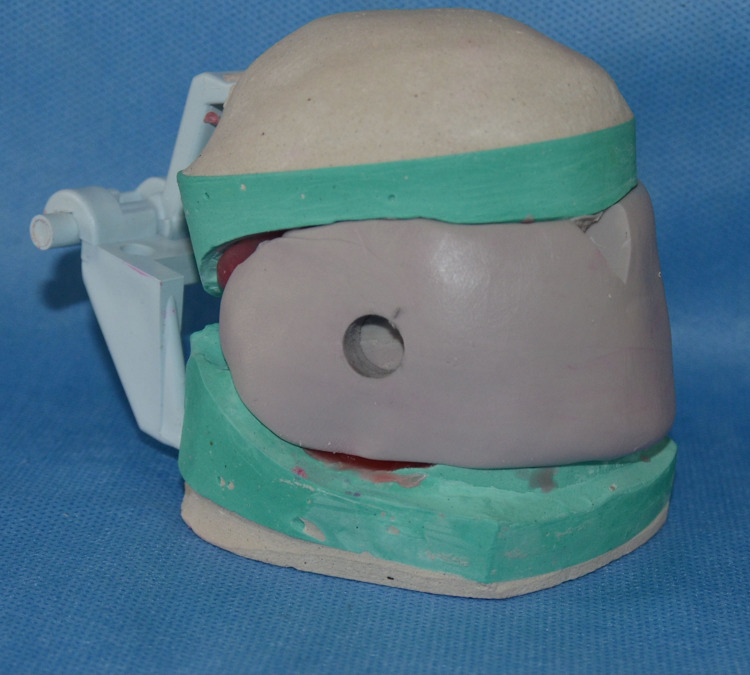
Putty index on labial and buccal surface with orientation grooves

**Figure 6:**
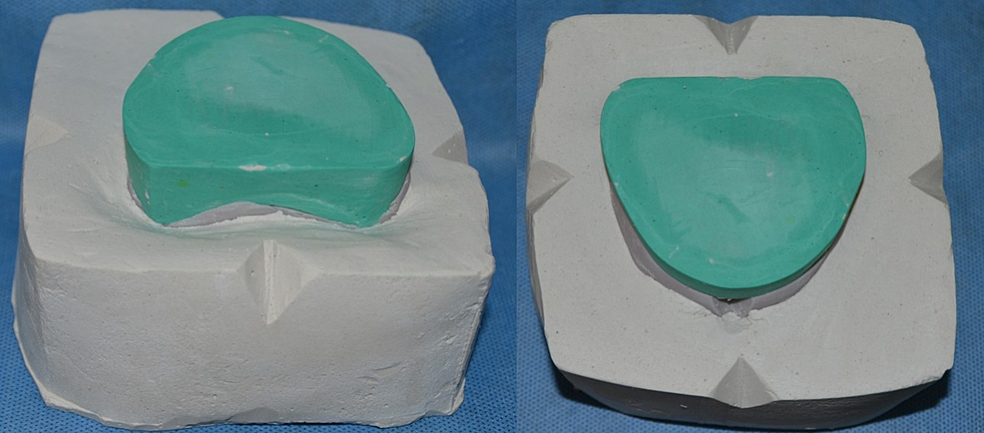
Flasking of the prosthesis with orientation grooves

Putty consistency addition silicone material was manipulated over the concave polished waxed surface of the MAD to facilitate ease of dewaxing. Orientation grooves were made over the addition silicone putty index on buccal and labial surfaces (Figure 5). The dimension of the casts warranted a customized flask that was fabricated using a cardboard with a lower and upper compartment. The first mix of type II gypsum was poured into the lower compartment, and the wax-up model was placed to cover the base of the mandibular cast. The second mix of type II gypsum was poured until three fourth of the putty index was covered and it was ensured that the maxillary cast was not immersed into the dental plaster (Figure 6). Orientation grooves were placed over the plaster in the lower compartment of the flask and a separating medium was applied. Flasking was completed by pouring the counter with type II gypsum in the upper compartment.

**Figure 7:**
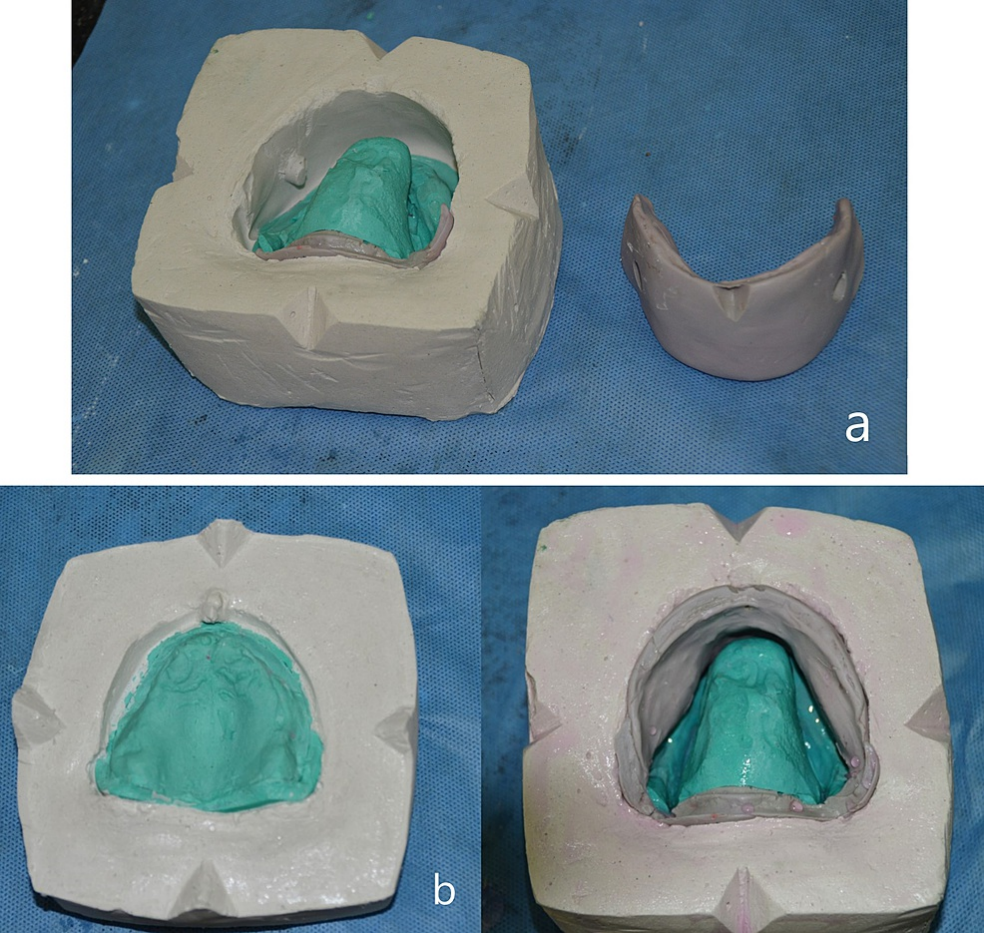
Putty index removed for wax elimination (a) and index reoriented (b)

**Figure 8:**
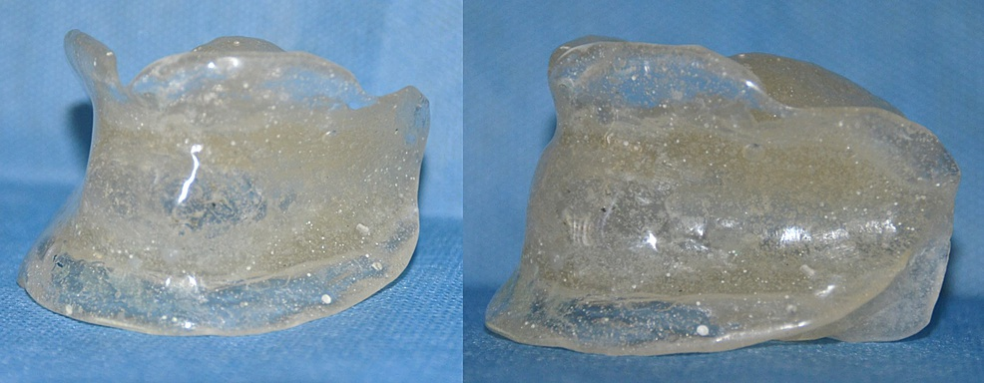
Completed prosthesis

Dewaxing of the flask resulted in the separation of the maxillary and mandibular moulds. The putty index was removed from the lower compartment of the flask mould. This enabled retrieval of the mandibular trial denture base (Figure 7a) and removal of residual wax beneath the tongue replica which would otherwise be locked within the undercut. The putty index was reoriented to the plaster wall of the flask body using the orientation grooves as a guide (Figure 7b). After application of the separating medium to the mould, heat cure clear denture base resin was packed and subjected to a long duration polymerization cycle. The final prosthesis was retrieved and the MAD was inserted after completion of trimming and polishing (Figure 8). The patient was instructed to position the tongue in the provided space at sleep. The patient was reviewed at 24 hours, one week, and every one month for a period of one year. The patient reported improvement in night sleep and reduction in daytime tiredness.

## Discussion

Continuous positive airway pressure being a gold standard treatment to open the upper airway by application of controlled compressed air is still not a widely accepted mode of treatment among OSA individuals due to difficulty in adaption to the equipment and high cost [6,7]. Management of mild to moderate OSA with oral appliances (OAs) is a better alternative to CPAP and surgical intervention [8-10]. The American Academy of Sleep Medicine has revised its indications and advocates oral appliance (OA) therapy as an alternative for adult patients with OSA who are intolerant to CPAP therapy. An OA therapy using MAD opens the airway by positioning the mandible forward and has become one of the alternative modalities of treatment. Prefabricated OAs like MAD, tongue holding devices reduced the chair-side fabrication time but had poor adaptation, increased salivation, and deprivation of sleep, and in addition, prolonged wear of ill-fit prefabricated prosthesis lead to temporomandibular joint (TMJ) pain and myofascial discomfort. Customized OA’s can overcome these challenges, but its adaptation also depends on patients’ oral health status, and the clinician’s skill.

The preliminary challenge was to obtain the retention of an OA in an edentulous situation at an optimum vertical dimension. Fabrication of MAD over the existing complete denture would require increasing the vertical dimension to a greater extent which might keep the muscle in a strained position and it was also observed to have no effect in improving the OA treatment [11]. Moreover, providing MAD at increased vertical position causes strain over the labial aspect of mandibular anterior and palatal aspect of maxillary anterior leading to shift in their occlusal relationship and symptoms of pain [12]. Hence, we chose the vertical dimension at rest as the vertical height of the device which would provide an unstrained position.

Loss of vertical dimension, hypertrophic tongue, and alteration in size of the soft palate due to prolonged edentulousness leading to reduced posterior airway space [2] needs to be addressed while fabricating MAD for an individual with OSA. Early literature on the OA for a completely edentulous patient suffering from OSA’s by Meyer and Knudson reported the use of MAD drastically decreased Apnea-Hypopnea Index (AHI) from 18.0 to 2.2/hour [13,14]. Nayar and Knox stated that the customized fabrication of MAD for an edentulous patient improved the retention and patient compliance, however, their assessment was based on subjective data without polysomnography [15]. The mechanism of action of the OA is through the advancement of the mandible which brings the tongue forward to increase the hypopharyngeal airway space [16]. To increase the airway space, we used 75% of the maximum amount of mandibular protrusion, since literature evidence suggests 75% of maximum protrusion was essential for the improvement in respiration with a reduced adverse effect on the temporomandibular joint and occlusal changes [17]. However, Marklund et al. proposed complete mandibular protrusion in severe OSA patients [18].

This clinical report differed from the MAD reported in the literature as it was designed to provide additional space for positioning the tongue anteriorly to synergize the effect of the MAD in an edentulous individual. The advancement of the mandible prevents the collapse of the posterior airway space by increasing the lateral dimension of the velopharyngeal area. A tongue retaining device positions the tongue anteriorly in the tongue space to create a “suction effect” that increases the velopharyngeal airway space when the patient is asleep [19]. Since prolonged edentulousness leads to the hypertrophied tongue, the presence of a denture pushes the tongue backward and narrows the posterior airway space [2]. Hence, the present clinical situation required a MAD that would hold the tongue in a forward position and provide an additive effect to the protruded mandible in improving the airway space.

The techniques for fabricating a customized MAD were not highly discussed in the literature. The custom-made prosthesis mentioned in this report described a one-piece monobloc design for mandibular advancement and tongue positioning device using a customized flask. The present clinical situation required a stable mandible at a defined position due to edentulousness that made us select the monobloc design. To reduce the weight of the monobloc design, and prevent discomfort to the patient, a concave external polished surface was provided. The undercut in the external and internal surfaces of the final wax-up model was a major challenge during flasking procedure. The putty indexing during flasking procedure ensured easy dewaxing below the undercut regions and enabled reorientation of the plaster mould that avoided mould fracture, especially when fabricating a monobloc design. We observed the patient did not have any complaints with the monobloc design comprising of MAD with a tongue holding space, during the follow-up period of one year. Literature states that the majority of difficulties faced by the patient were within the first year, and that was not observed in our case [20]. The limitation of the case report was that it cannot be generalized to the population. However, the positive outcome observed without any prosthesis-related complications can be attributed to the dual-functional appliance provided at an unstrained vertical dimension and 75% of maximum protrusion. This case report has provided a complete insight into the clinical and technical view in the fabrication of the MAD with tongue space for an edentulous individual.

## Conclusions

Management of sleep apnoea is a challenging task, especially in an edentulous individual. The loss of vertical dimension, hypertrophy of the tongue plays a major role in accentuating the sleep disturbances. Management of individuals suffering from OSA and non-compliance with CPAP devices have paved the way for alternative treatment with removable appliances. The clinical and technical aspects described in this report with customized MAD with tongue holding space would help the patient achieve patency of the airway, and thereby improve his/her quality of sleep. The appliance restores the adequate vertical dimension, provides adequate retention and stability due to the monobloc design, and moves both the mandible and tongue forward to provide adequate posterior airway space and thereby decreasing sleep apnoea.

## References

[1] Heidsieck DS, de Ruiter MH, de Lange J (2016). Management of obstructive sleep apnea in edentulous patients: an overview of the literature. Sleep Breath.

[2] Padmanabhan TV, Banu RF, Mahalakshmi A, Aziz A, Bohra S, Kumar VA (2015). Dimensional change in soft tissues with complete dental prosthesis and its effect on airway space and natural head position. Indian J Dent Res.

[3] Jayesh SR, Bhat WM (2015). Mandibular advancement device for obstructive sleep apnea: an overview. J Pharm Bioallied Sci.

[4] Yoshida K (2000). Effects of a mandibular advancement device for the treatment of sleep apnea syndrome and snoring on respiratory function and sleep quality. Cranio.

[5] Marklund M, Verbraecken J, Randerath W (2012). Non-CPAP therapies in obstructive sleep apnoea: mandibular advancement device therapy. Eur Respir J.

[6] Sutherland K, Vanderveken OM, Tsuda H, Marklund M, Gagnadoux F, Kushida CA, Cistulli PA (2014). Oral appliance treatment for obstructive sleep apnea: an update. J Clin Sleep Med.

[7] Sutherland K, Phillips CL, Davies A (2014). CPAP pressure for prediction of oral appliance treatment response in obstructive sleep apnea. J Clin Sleep Med.

[8] Kushida CA, Morgenthaler TI, Littner MR (2006). Practice parameters for the treatment of snoring and Obstructive Sleep Apnea with oral appliances: an update for 2005. Sleep.

[9] Lim J, Lasserson TJ, Fleetham J, Wright J (2006). Oral appliances for obstructive sleep apnoea. Cochrane Database Syst Rev.

[10] Chan AS, Cistulli PA (2009). Oral appliance treatment of obstructive sleep apnea: an update. Curr Opin Pulm Med.

[11] Pitsis AJ, Darendeliler MA, Gotsopoulos H, Petocz P, Cistulli PA (2002). Effect of vertical dimension on efficacy of oral appliance therapy in obstructive sleep apnea. Am J Respir Crit Care Med.

[12] Ghazal A, Jonas IE, Rose EC (2008). Dental side effects of mandibular advancement appliances - a 2-year follow-up. J Orofac Orthop.

[13] Meyer JB Jr, Knudson RC (1990). Fabrication of a prosthesis to prevent sleep apnea in edentulous patients. J Prosthet Dent.

[14] Giannasi LC, Magini M, de Oliveira CS, de Oliveira LV (2008). Treatment of obstructive sleep apnea using an adjustable mandibular repositioning appliance fitted to a total prosthesis in a maxillary edentulous patient. Sleep Breath.

[15] Nayar S, Knox J (2005). Management of obstructive sleep apnea in an edentulous patient with a mandibular advancement splint: a clinical report. J Prosthet Dent.

[16] Wang W, Di C, Mona S, Wang L, Hans M (2018). Tongue function: an underrecognized component in the treatment of obstructive sleep apnea with mandibular repositioning appliance. Can Respir J.

[17] Ringqvist M, Walker-Engström ML, Tegelberg A (2003). Dental and skeletal changes after 4 years of obstructive sleep apnea treatment with a mandibular advancement device: a prospective, randomized study. Am J Orthod Dentofacial Orthop.

[18] Marklund M, Franklin KA, Sahlin C, Lundgren R (1998). The effect of a mandibular advancement device on apneas and sleep in patients with obstructive sleep apnea. Chest.

[19] Sutherland K, Deane SA, Chan AS, Schwab RJ, Ng AT, Darendeliler MA, Cistulli PA (2011). Comparative effects of two oral appliances on upper airway structure in obstructive sleep apnea. Sleep.

[20] Doff MH, Veldhuis SK, Hoekema A, Slater JJ, Wijkstra PJ, de Bont LG, Stegenga B (2012). Long-term oral appliance therapy in obstructive sleep apnea syndrome: a controlled study on temporomandibular side effects. Clin Oral Investig.

